# Contralateral diaphragmatic injury sustained from transhumeral amputation: an unusual case and brief literature review

**DOI:** 10.1093/jscr/rjac346

**Published:** 2022-07-30

**Authors:** Vincent Marcucci, Stuart Campbell, Emmanuel Ihionkhan, Ajul Shah, Thomas Bauer, Abimbola Pratt

**Affiliations:** Department of Surgery, Jersey Shore University Medical Center, Neptune, NJ, USA; Department of Surgery, Jersey Shore University Medical Center, Neptune, NJ, USA; School of Medicine, Hackensack Meridian University, Hackensack, NJ, USA; Department of Surgery, Jersey Shore University Medical Center, Neptune, NJ, USA; School of Medicine, Hackensack Meridian University, Hackensack, NJ, USA; Department of Surgery, Jersey Shore University Medical Center, Neptune, NJ, USA; School of Medicine, Hackensack Meridian University, Hackensack, NJ, USA; Department of Surgery, Jersey Shore University Medical Center, Neptune, NJ, USA; School of Medicine, Hackensack Meridian University, Hackensack, NJ, USA

**Keywords:** diaphragmatic injury, thoracoscopy, case report

## Abstract

Right-sided diaphragmatic injury is an uncommon sequelae from blunt trauma and may be associated with other severe thoracoabdominal injuries. This injury can be easily missed on initial assessment and a high index of suspicion and clinical judgment is required. Recently, we treated a 25-year-old male inflicted with a right-sided diaphragmatic injury after a left-sided transhumeral amputation sustained from an overturned motor vehicle collision with thoracoscopic exploration and reapproximation.

## INTRODUCTION

Diaphragmatic injuries are uncommon and include wounds or ruptures that occur as a result of blunt or penetrating trauma to the thorax and/or abdomen. Preoperative diagnosis of blunt traumatic diaphragmatic injuries is quite difficult because it is often accompanied by other injuries in the chest, abdomen and extremities [1]. The left hemidiaphragm is more frequently damaged when compared with right-sided diaphragmatic injury; largely due to the protective effect of the liver [2, 3]. Most blunt right-sided diaphragmatic injuries are due to high-speed motor vehicle accidents and falls from significant height [4]. The mechanism of injury in diaphragmatic ruptures remains undetermined; however, several theoretical causes have been investigated [4]. Surgical repair via laparoscopic and thoracoscopic approaches have been increasingly utilized; however, an open abdominal approach remains the mainstay for operative repair [5].

### CASE PRESENTATION

**Figure 1:**
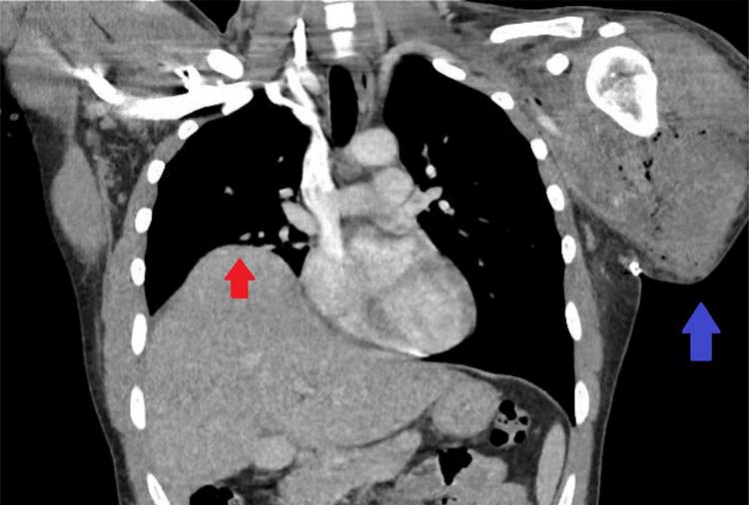
CT scan of the chest showing right-sided elevation/eventration of the diaphragm concerning for phrenic nerve injury (Red arrow); post-operative changes to LUE stump (Blue arrow).

We present a case of a 25-year-old male who sustained a traumatic left-sided transhumeral amputation in a single car roll-over motor vehicle collision (MVC). The patient was transferred to a tertiary care center under the care of the plastic surgery service for further management and plans for operative reinnervation of his left upper extremity (LUE). Upon arrival, the patient’s only complaint was discomfort to the LUE stump. He was subsequently pan-scanned with a computed tomography (CT) due to the mechanism of the crash sustaining significant blunt abdominal trauma. The patient was found to have elevation of his right hemidiaphragm, concerning for possible diaphragmatic injury demonstrated in Fig. 1. A fluoroscopic sniff test was completed showing no inferior or superior excursion of the right diaphragm during inspiration or expiration with paradoxical chest wall motion providing diagnostic evidence of right hemi-diaphragmatic paralysis.

After successful muscle nerve neurolysis and extensive nerve transfer to the LUE, the patient underwent a diagnostic laparoscopy, diagnostic thoracoscopy and chest tube insertion in the right pleural space. The right hemi-thorax was evaluated and a grade 3 right diaphragmatic injury based on the American Association for the Surgery of Trauma with extension of the hiatus of vena cava lateral to falciform was identified, as well as a grade 1 liver injury-capsular tear. The remainder of the peritoneal cavity was examined, as well as the left hemi-thorax, which did not show any signs of injury. Postoperatively, the patient was incidentally diagnosed with COVID-19. He was not complaining of any respiratory symptoms at this time; his only complaint was discomfort at the chest tube insertion site and to his LUE stump. His oxygen saturation remained at 99–100% on room air during his hospital course.

**Figure 2:**
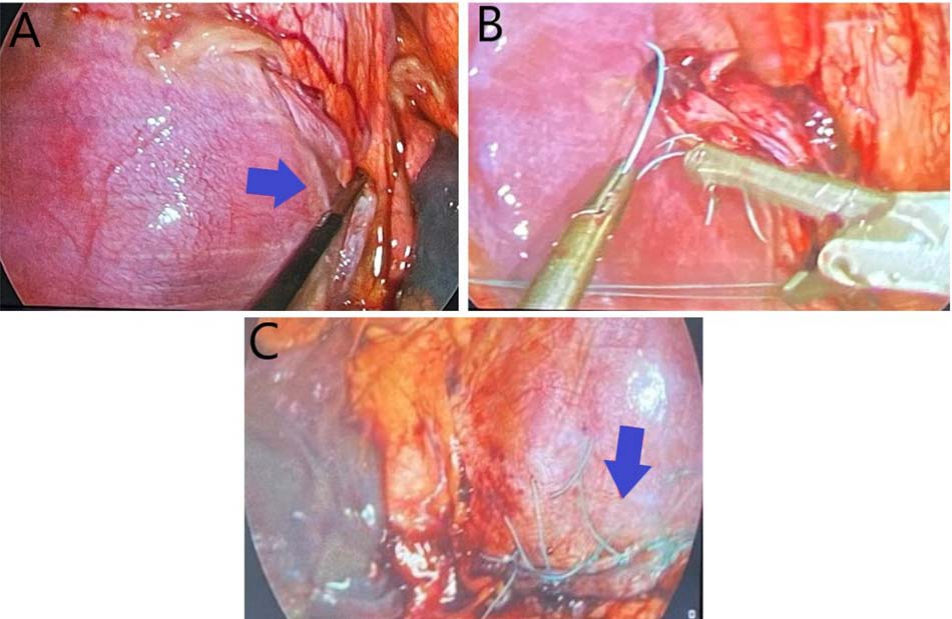
(**A**) Demonstrates a right hemi-diaphragmatic injury; (**B**) Depicts thoracoscopic suturing of the diaphragmatic defect; (**C**) shows completed thoracoscopic repair of the diaphragmatic injury.

**Figure 3:**
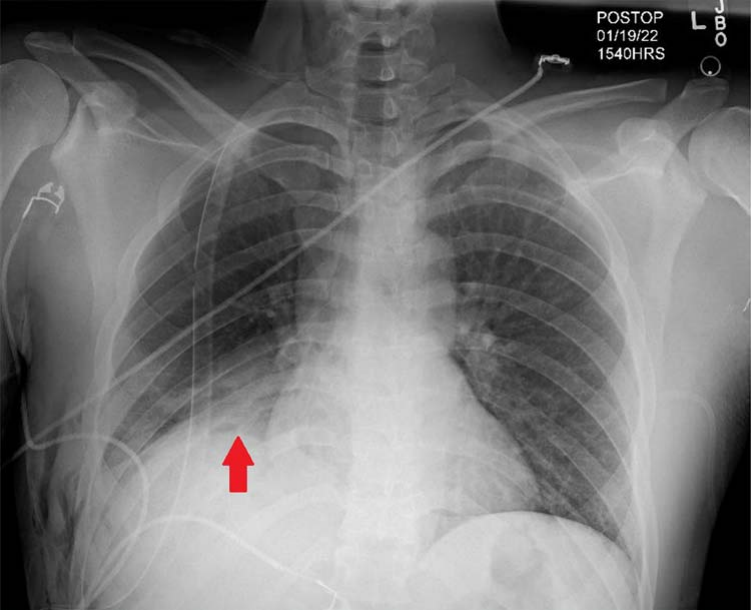
Post-operative CXR demonstrating appropriate placement of a thoracoscopic tube and improvement of right-hemi-thorax eventration (Red arrow).

Days later, the patient was brought back to the operating theater for definitive repair of his diaphragmatic injury shown in Fig. 2A. Nonabsorbable sutures were utilized in a horizontal mattress technique to reapproximate the diaphragm thoracoscopically depicted in Fig. 2B and C, successfully reducing the liver into the abdomen. The phrenic nerve was identified and found to be intact prior to and after repair of the diaphragmatic injury. The patient tolerated the procedure well without complication. A postoperative chest X-ray (CXR) was done showing expansion of the right hemi-thorax compared with preoperative imaging as displayed in Fig. 3. On postoperative day (POD) #1, the patient’s thoracoscopic tube was taken off of suction and removed. His vital signs remained stable during his postoperative course with appropriate pain control; he was discharged home on POD#2.

## DISCUSSION

The diaphragm is a dome-shaped layer of skeletal muscle with a central portion of fibrous tissue that functions as the primary respiratory muscle, as well as separating the thoracic cavity from the abdominal cavity [6]. Traumatic diaphragmatic rupture(s) are relatively uncommon but can be seen with severe blunt thoracoabdominal or penetrating injury. In a 2012 study, consisting of over 800 000 traumas from the American College of Surgeons National Trauma Data Bank found the overall incidence of diaphragmatic injury to be ~0.46%; with more than two-thirds sustained from penetrating trauma [7]. The most common mechanism of blunt traumatic injury to the diaphragm was from an MVC, comprising over 60% of the cases [7, 8]. Diaphragmatic injury is more commonly seen on the left side because the liver functions as a protective barrier to the right hemidiaphragm, with an incidence of right-sided injury in nearly 13% of cases [6]. Injury to the diaphragm is frequently identified along the posterolateral aspect of the hemidiaphragm as a result of embryological weakness [6]. Mechanism of injury may be caused by shearing of a stretched membrane and avulsion of the diaphragm from its point of attachment, sudden force transmission through the viscera or increased abdominal pressure exceeding the bursting pressure of the diaphragm [4]. Referred to as deceleration injuries, the production of shearing forces during rapid deceleration in motor vehicle accidents cause direct compression against fixed points leading to diaphragmatic rupture and tracheobronchial disruption [9].

Prompt and accurate diagnosis of penetrating and blunt diaphragmatic injury can be challenging for radiologists and surgeons even with the help of advanced diagnostic techniques [8]. CT scan remains the gold standard for diagnosis of diaphragmatic rupture. Sonography has been a useful diagnostic tool, in particular focused abdominal sonography for trauma with close attention to diaphragmatic motion artifact and hemothorax [10]. As in our patient, his initial CT scan of the chest prior to transfer to our hospital did not show the signs of diaphragmatic injury. The patient at this time was also asymptomatic. It was not until after diagnostic laparoscopy that revealed a diaphragmatic injury with concomitant liver laceration. It is uncertain if the shearing force from the overturned vehicle and transhumeral amputation or blunt trauma caused the diaphragmatic injury in our case.

In regards to the treatment of right-sided diaphragmatic rupture, midline laparotomy has been the recommended surgical approach, largely because it allows for the exploration of the peritoneal cavity [10]. In our case, the patient remained hemodynamically stable prior to definitive repair and underwent diagnostic laparoscopy and thoracoscopy allowing for isolated repair of the diaphragmatic injury. Repair of the diaphragmatic defect utilizes non-absorbable sutures in an interrupted or continuous fashion with placement of a chest tube within the pleural space [11]. Video-assisted thoracoscopic surgery has a higher sensitivity and specificity when compared with laparoscopy and has been shown to reduce the risk of tension pneumothorax [1, 11].

Diaphragmatic injury-related mortality is low and typically caused by the concomitant injuries suffered during an isolated traumatic event. In patients with documented diaphragmatic injury, shock, traumatic brain injury and organ failure have been the leading causes of death [12]. The timeline of recovery and overall outcomes for patients who have undergone repair for a diaphragm rupture is highly correlated with the severity of associated injuries and co-morbid conditions [12]. It has been found that delayed and/or inaccurate diagnosis may increase mortality by up to 30% [10, 13].

In conclusion, right-sided diaphragmatic injury from blunt trauma is an uncommon finding and can cause difficulty in accurately diagnosing the injury in a timely manner. Particular attention should be paid to patients who have suffered blunt trauma from an MVC, especially from a frontal or lateral impact. The diagnostic algorithm including radiography and clinical suspicion should be applied with patients who have suspected thoracoabdominal injury.
